# Community-based health insurance and social capital: a review

**DOI:** 10.1186/2191-1991-2-5

**Published:** 2012-04-04

**Authors:** Hermann Pierre Pythagore Donfouet, Pierre-Alexandre Mahieu

**Affiliations:** 1 CREM, UMR CNRS 6211, University of Rennes I, 7 Place Hoche, 35 065 Rennes Cedex, France; 2 LEMNA, University of Nantes, Chemin de la Censive du Tertre, 44 322 Nantes Cedex, France

**Keywords:** Community-based health insurance, Social capital, Rural areas

## Abstract

Community-Based Health Insurance (CBHI) is an emerging concept for providing financial protection against the cost of illness and improving access to quality health services for low-income rural households who are excluded from formal insurance. CBHI is currently being provided in some rural areas in developing countries and there is ongoing research about its impact on the well-being of the poor in these areas. However, the success of CBHI revolves around the existence of social capital in the community. This has led researchers to explore the impact of CBHI on the well-being of the poor in rural areas, especially as it relates to social capital. The overall objective of this paper is to review recent developments that address the link between CBHI and social capital. Policy implications are also discussed.

**JEL Classification:**

C10, I15

## Introduction

Financing quality health care is a major challenge in developing countries. Health plays a key role in the economic development of a country. For this reason, policy-makers in developing countries have increased their efforts towards providing quality health care both in urban and rural areas [1,2]. Despite these efforts, many countries still have low geographical coverage quality health care. For Example, in Sub-Saharan African countries, inhabitants are facing precarious health conditions. About 26 per cent of children under five years old are underweight [3]. According to Morrisson *et al. *[4], the percentage of children suffering from acute malnutrition (and whose families are classified below the absolute poverty threshold) ranged from approximately 15 to 20 per cent in intermediate revenue countries (Cameroon, Ivory Coast, Zimbabwe), to more than 50 per cent in very poor countries such as Madagascar and Niger. About 42 million children below five years old suffered from acute malnutrition in Sub -Saharan Africa in 1996. The most recent estimates released by the United Nations Food and Agriculture Organization (UNFAO) [5] shows that the majority of the world's undernourished people live in developing countries. In the world, 925 million people are undernourished, of which 239 million live in Sub-Saharan Africa countries. This malnutrition will continue to cause poor health and affect the well-being of the households in this region of the World if adequate measures are not taken.

Over the last years, demographic growth coupled with unequal growth in these regions has increased the number of people who are living in extreme poverty. Nowadays, about 50 per cent of the people in Sub-Saharan Africa are living below the poverty threshold. Furthermore, more than 100 million people do not have a balanced diet [6]. Such a situation increasingly affects rural areas in developing countries which have very low standards of well-being [7] and quality health care [8]. Most households in these rural areas are characterised by a high prevalence rate of sanitation-related diseases, which undermines their health, in turn weakening their ability work and invest.

The disappearance of free health care (mostly primary health care) has resulted in the loss of a form of social protection for a large portion of the population especially rural households and those working in the informal sector. As a result, many policy-makers, international institutions, NGOs and the civil society have set out to seek effective alternatives in order to provide rural households a permanent solution to the problem of accessing health care. Development actors are increasingly considering community-based health insurance (CBHI)^1 ^as an instrument that can enable not only easy access to quality health care, but also reduce absolute poverty among low-income populations. CBHI is a form of micro health insurance which is mainly used in rural areas in developing countries [9-11]. Over the last decades, insurance was recognized as a financial instrument which could enable low-income households to manage their financial risks [11]. The role of CBHI therefore is to help low-income households manage risks and reduce their vulnerability in the face of financial shocks. CBHI is usually based on the following characteristics: voluntary membership, non-profit objective, linked to a health care provider (often a hospital in the area), risk pooling and relying on an ethic of mutual aid/solidarity [12], p.5. According to Churchill [13], p.12, CBHI: *"*refers to the protection of low-income people against specific perils in exchange for regular premium payments proportionate to the likelihood and cost of the risk involved". Several studies show that low-income households are willing to pay for CBHI [9,14,15]. In most cases, the payment of the premiums are in cash (monthly, quarterly, yearly) or in kind [16] such as agricultural commodities. However, the success of CBHI relies on the existence of social capital in the community. As declared by Woolcock and Narayan [17], p.3: "social capital refers to the norms and networks that enable individuals to act collectively". Fukuyama [18], p.4 asserted that "social capital can be defined simply as the existence of a certain set of informal values or norms shared among the members of a group that permit cooperation among them". Sobel [19] describes social capital as circumstances in which individuals can benefit from group membership. Thus, social capital refers to social life-networks, norms, and trust that enables households to act together more effectively to pursue shared objectives [20,21]. This social capital in the community can be an asset for the breakthrough of CBHI, thus contributing to the demand for CBHI at the community level. As outlined by the BIT [22], one of the key principles of a good functioning of CBHI is the solidarity and trust between members. This solidarity and trust stirs up members who are susceptible to risk to put together their resources for common use. Hence, the overall objective of the paper is to review the link between CBHI and social capital. The remainder of the paper is organized as follows; section 2 focuses on recent developments that address the link between CBHI and social capital, section 3 presents a theoretical framework that shows the link between CBHI and social capital, and section 4 concludes with some policy implications.

## Recent works on CBHI and social capital

CBHI emanates from the limitations of the loan-based microcredit programs (microfinance) in protecting low-income households from health shocks. CBHI differs from other forms of microfinance in that it uses an insurance mechanism, i.e. a financial instrument which, in return for payment of a premium, provides members with a guarantee of financial compensation or service on the occurrence of specified events [23], p.13. Unlike microfinance where transfer in the first instance takes place from the credit provider to the poor, in case of CBHI a reverse transfer takes place, i.e., from the poor to the insurance provider [12], p.2. The success of CBHI depends on the existence of social capital in the community.

The concept of social capital has been a motivation for decision-makers to reflect on issues of health improvement. Many international organisations such as the OECD, the World Bank, the UNESCO, have emphasized on social capital, which they consider as a powerful tool for attaining the objectives of development actors both in developed and developing countries. In the 90s, one of the main goals of the World Bank was to use the potential of social capital to fight poverty and ensure availability and access to health, banking facilities and education. CBHI can only be effective and long-lasting with the aid of social capital in a community, as social capital has a positive effect on the community's demand for insurance. As outlined by the BIT [22], one of the key principles for the good functioning of a CBHI is the solidarity and trust between the members. Solidarity and trust stirs up members who are susceptible to risk to put together their resources for common use. In empirical analyses, ignoring the role of social capital in WTP may result in omitted variable bias, as the decision to pay for CBHI may be correlated with variables which are not included in the model. As a matter of fact, social capital in a community is vital for the sustainability and effective functioning of CBHI [24,25]. In some areas of Africa such as Duekoué (Ivory Coast), post electoral violence has led to the fragmentation and lack of trust. Therefore, it will be difficult to establish CBHI in that community.

Authors such as Coleman [21], Putnam *et al. *[26], Wilkinson [27], unanimously acknowledge that social capital in a community acts positively on the importance people attach to their health. Thus, a community with a high level of social capital will be more inclined to go through change. Therefore, more ready to support a new, unknown health policy such as CBHI. Consequently, adhesion to a group and trust are necessary to enable poor communities establish social capital and have access to CBHI. On the one hand, if connections are lacking, or if the level of social capital among the members of the group is weak, there is an increasing risk of seeing egoistic behaviour as the highest levels of moral risk and anti-selection. In addition, Baum [28], Kawachi *et al. *[29] demonstrate that a high level of trust in the community will facilitate cooperation, aids, access to health care. Thus acting together, low-income households will have opportunities to increase their income and social well-being [30]. As outlined by Woolcock and Narayan [17], social capital helps the poor to manage risk and vulnerability. Thus, CBHI which aims at managing risk and vulnerability may be well accepted by a community that possesses a high stock of social capital. A high level of social capital is associated with a high level of altruism among individuals; this makes it possible to take into consideration the well-being of other members of the group [31]. As outlined by Hsiao [32], p.5, social capital is a major determinant of the WTP for CBHI, the greater the social capital in the community, the more people are willing to prepay for CBHI. In his study, the author considers two communities. Community A has less social capital than community B. Thus, community B will have the greater potential of the establishment and success of CBHI than community A. He further concludes that community A will not be able to establish CBHI since there is a low level of social capital in that community. Furthermore, CBHI will be successful in community B even if the prepayment amount is greater than the expected economic/health gain because of the social capital in that community. Through his analysis, policymakers may understand why some CBHI have been successful while others have failed in developing countries.

Recently, Zhang *et al. *[24] explored the effect of social capital on the demand for CBHI subsidized by the Chinese government. The Government aimed at encouraging Chinese farmers in villages to join CBHI companies by subsidizing the annual allowance of each participant by 10-20 Yuan (1.25-2.50 US $). Trust and reciprocity were used as proxies of social capital to obtain the effect of social capital on the demand (WTP). Ten questions were asked to assess the effect of social capital on WTP (five questions on trust and five others on reciprocity). The answers to these questions ranged between "I agree" to "I disagree". Five questions were on horizontal trust (trust in community members) and not on vertical trust (trust in the government, municipal authorities, etc.). The questions were: "Do you think a majority of the villagers can be trusted? Do you think the villagers in your community can consider you to attain their goals if they had the opportunity? Do you think the villagers in your community can restore lost objects they have found to the rightful owners? Do you think a majority of your neighbours are trustworthy? Do you think the leaders of your village can be trusted?" The five questions on reciprocity were: "Do you think the villagers are concerned with problems that do not concern them, but that concern other villagers? Do you think villagers would help someone in need? Would you lend money to your neighbour if he needs to consult a doctor? If your village were a big family, would you be a member of this family? Would you support projects that do not benefit you, but benefit other inhabitants of your village?" Using logistic regression, the results of such a study demonstrate that social capital measured by trust and reciprocity has a positive and significant effect on the demand for CBHI.

In another recent study, Donfouet *et al. *[25] investigated the link between CBHI and social capital in rural Cameroon (Central Africa). The study is based on a face-to-face survey. Over 399 households across six villages in Bandjoun (Western Region of Cameroon) were selected by a two-stage cluster sampling technique. The six villages were: Tsela, Mbiem, Mbouo, Pète, Dja and Toba. Firstly, six villages were selected-based on population size and availability of health centres. Secondly, household heads in these villages were randomly selected. Guidelines for a rigorous contingent valuation^2 ^provided by Whittington [33], Arrow *et al. *[34], and Carson (2000) were followed as much as possible. Furthermore, to mitigate hypothetical bias,^3 ^an *ex-ante *approach (the budget reminder and the consequentialism script) was integrated in the questionnaire. The social capital was measured as the degree of involvement of households in associations. By using an interval regression model of Cameron [35], the results of the study confirmed that social capital has a positive, and significant impact on the demand for CBHI.

Policy makers might want to increase social capital, but an open question is what type of social capital to increase. The two main types of social capital are often labelled "weak ties" (also called "bridging social capital") and "strong ties" (also called "bonding social capital"). According to Woolcock and Narayan [17], p. 8, "strong ties" refers to the close relationship between an individual and his family, friends, ethnic group, etc. This corresponds to intra-community social capital. "Weak ties" refers to the individual's contacts outside the ethnic group or the family (other entrepreneurs, other ethnic groups, banks, etc.). This corresponds to extra-community social capital. In other words, "strong ties" refers to the interactions that exist within a particular group (closed family, friends), whereas "weak ties" refers to the interactions across multiple groups (open groups or networks). Each type of social capital has pros and cons and it is unclear which one ought to be reinforced in priority. "Strong ties" implies a high level of solidarity between the members of the group, which is good for CBHI. However, similitude between members is a flaw for CBHI. For example if all members undertake risky behaviour, CBHI might not work properly. On the other hand, "weak ties" implies less solidarity, but members are different from each other. More risky behaviour might be compensated by less risky behaviour. Further studies could investigate which type of social capital has the strongest effect on CBHI. If "weak ties" works better, policy makers might, for instance, decide to start developing CBHI in bigger villages, since the level of extra-community social capital is high.

It should be noted that, though, social capital could significantly affect a households' decision for health insurance, up to date, there is no clear consensus on how social capital should be measured. As stated by Fukuyama [18], p.12: *"one of the greatest weaknesses of the social capital concept is the absence of consensus on how to measure it"*.

## Theoretical framework

Popularized by Putnam [20], the concept of social capital has been the subject of several studies that have attempted to measure it. Some of these studies treat social capital as a dependent variable (a phenomenon which has to be explained), and this is the institutional view of social capital that argues that the vitality of community networks and civic society is largely the product of the political and institutional environment [17,36]. An alternative method, developed in recent years is to study the role or function, or better still, the contribution of social capital to health. Therefore, social capital is studied as an independent variable (this is a communitarian view of social capital). The issue is thus to bring out its contribution to access to basic health care for rural households. Its scope is basically social networks, trust or associations which can improve the health situation of households. Therefore, a positive relationship between CBHI and social capital is expected.

Let Y be the dependent variable, such as the willingness-to-pay (WTP) for CBHI, X_1_, *X*_2 _....X_n _the independent variables, and a random component. Y is a linear function of the explanatory variables:

(1) $$Y=\beta_{o}+\beta_{1}X_{1}+\overset{n}{\underset{i=2}{\sum }}\beta_{i}X_{i}+\varepsilon$$

In equation (1), one of the explanatory variables, say X_1_, refers to social capital and the others are related to socio-demographic characteristics such as age, gender, income, marital status, number of children in the households, profession etc.

Depending on how Y is measured, a specific estimation model can be used. For instance, if Y is a continuous variable, the ordinary least squared (OLS) model is used. If Y is a binary variable, the probit or logit model is used. If Y lies in interval, the interval regression is used [35].

The partial derivative of Y with respect to X_1 _is:

(2) $$\frac{\partial Y}{\partial X_{1}}=\beta_{1}$$

If *β*_1 _is positive and statistically significant at conventional levels, there is a positive relationship between the WTP for CBHI and social capital. Theoretically and empirically, *β*_1 _must be positive since it is always expected that solidarity, norms, trust and participation at the community level to have a positive effect on the demand for CBHI.

## Conclusions

Access to health services is a main concern in poor countries. Most policy debates are around how to keep the poor from falling into a poverty trap that is often caused by medical expenses. Delaying medical treatment or choosing self-treatment can generate serious health consequences. Hence tackling this issue is of utmost importance. Many policymakers in developing countries are trying to develop health care programs that would cater to the poor and be sustainable at the same time.

Lack of health insurance coverage of the poor in developing countries impedes access to adequate health care. Consequently, CBHI has been considered as an effective means to reach the poor with health care services. Since there has been an increased attention to such a health insurance scheme, the analysis of the demand for CBHI is extremely important for formulating policies and strategies for the health sector. Adequate knowledge of the determinants of healthcare demand is essential for devising strategies to increase allocative efficiency of resources. Nevertheless, for CBHI to have a long-term effect, there must be a social capital in the community. Thus, the overall objective of the study was to review recent developments that address the link between CBHI and social capital. A thorough review reveals that a higher level of social capital at the community level will positively and significantly impact households' decision for health insurance, which will in return increase the demand for CBHI. One important policy implication is to strengthen social ties at the community level. An open question for further studies is what type of social capital to develop in priority. Both inter and extra community social capital has pros and cons, and it is unclear which one ought to be reinforced.

## Endnotes

^a ^It is also called community health funds, mutual health organizations, rural health insurance, etc.

^b ^Contingent valuation is a survey-based method used to assess the value participants attach to public goods which are not provided by the market. It is commonly used in the health, marketing, education and environment sectors.

^c ^The hypothetical bias is the difference between the hypothetical WTP and the real WTP.

## Competing interests

The authors declare that they have no competing interests.

## Authors' contributions

The authors did the research jointly. All authors read and approved the final manuscript.
